# From the Ear to the Brain: Otitis Externa Complicated by Coalescent Mastoiditis Leading to Temporal Lobe Abscess and Wernicke’s Aphasia

**DOI:** 10.7759/cureus.99057

**Published:** 2025-12-12

**Authors:** Sujatha Sekar, Madhu Murali, Divyaradha Krishnan Seethapathy, Annapoorna Nair, Diorella Lopez- Gonzalez

**Affiliations:** 1 Department of Family Medicine, University of Arkansas for Medical Sciences, Little Rock, USA

**Keywords:** central nervous system infection, diabetes mellitus, family medicine, intracranial infection, mastoiditis, otitis externa, otogenic brain abscess, tegmen tympani defect, temporal lobe abscess, wernicke’s aphasia

## Abstract

Otitis externa is typically a benign infection of the external auditory canal; however, in high-risk individuals, particularly those with poorly controlled diabetes mellitus, it may progress to severe otogenic and intracranial complications. We report a case of a 59-year-old woman with type 2 diabetes and an HbA1c of 14%, reflecting longstanding uncontrolled disease related to medication nonadherence, who developed a temporal lobe abscess and Wernicke’s aphasia following inadequately treated otitis externa. At her initial emergency department (ED) visit for left-sided otalgia, otoscopic examination demonstrated erythema and crusting of the external auditory canal with an intact tympanic membrane, preserved hearing, and no mastoid tenderness. She was prescribed topical and oral ciprofloxacin but discontinued both after one day due to vomiting. Laboratory studies revealed marked hyperglycemia with otherwise normal inflammatory markers. Two weeks later, she returned with confusion, disorientation, and fluent but nonsensical speech. A CT angiogram performed for concern of acute stroke was unremarkable, while an MRI of the brain demonstrated complete opacification of the left mastoid and middle ear cavity, coalescent mastoiditis with erosion of the tegmen tympani, and a 9 × 5 × 7 mm peripherally enhancing lesion in the left middle temporal gyrus consistent with a temporal lobe abscess. CT of the temporal bone confirmed acute mastoiditis, although intracranial findings were better delineated on MRI. Blood cultures remained negative. Given the small size of the abscess and absence of mass effect, she was managed nonoperatively with intravenous cefepime and vancomycin and oral metronidazole, alongside intensive glycemic optimization. She completed a six-week antimicrobial regimen and achieved full neurological recovery with near-complete radiographic resolution. This case highlights that inadequately treated otitis externa may serve as a suspected otogenic source of intracranial abscess in patients with uncontrolled diabetes and emphasizes the importance of early MRI evaluation when neurologic symptoms arise, even when CT imaging and laboratory findings are initially reassuring.

## Introduction

Otitis externa, commonly referred to as “swimmer’s ear,” is an infection of the external auditory canal. Although typically benign, intracranial complications may occur, particularly when neurologic manifestations are subtle or nonspecific [1]. Early imaging is therefore essential when symptoms evolve atypically or worsen, consistent with radiologic recommendations for evaluating suspected intracranial complications of inflammatory ear disease [2]. Brain abscesses carry significant morbidity, and prompt recognition and management are critical to optimizing outcomes [3].

Progression from otitis externa to deeper infection is uncommon but has been reported, especially when the disease is untreated or inadequately treated [4]. Otogenic infections can extend medially from the external auditory canal into the middle ear and mastoid. As inflammation intensifies, mastoid air cells may coalesce, and bone erosion can occur. The tegmen tympani, a thin bony plate separating the mastoid from the intracranial compartment, is particularly vulnerable. When this barrier is breached, infection may spread into the middle cranial fossa and seed the adjacent temporal lobe, resulting in abscess formation [5,6]. Intracranial complications of otitis media and mastoid disease continue to be observed despite modern antimicrobial therapy [7]. Temporal lobe abscesses arising from contiguous spread of mastoid disease also remain well documented in contemporary series [8,9].

In diabetic patients, the differential diagnosis for severe otalgia and persistent symptoms includes necrotizing otitis externa, a skull base infection capable of aggressive extension. Diabetes further increases susceptibility to severe infections due to impaired immune responses and altered host defenses [10].

We present a case of a temporal lobe abscess and Wernicke’s aphasia in a patient with uncontrolled diabetes following inadequately treated otitis externa, highlighting that new aphasia or altered cognition after a recent ear infection should prompt early MRI, even when CT findings and routine laboratory values are initially reassuring.

## Case presentation

A 59-year-old woman with type 2 diabetes mellitus and hypertension presented with three to four days of altered mental status, confusion, and forgetfulness. Two weeks prior, she had been evaluated in the emergency department (ED) for left-sided otalgia. At that visit, otoscopic examination demonstrated erythema and crusting of the left external auditory canal, an intact tympanic membrane, normal hearing, and no mastoid tenderness. She was diagnosed with otitis externa and prescribed oral and topical ciprofloxacin, but she discontinued both medications after one day due to vomiting, resulting in an inadequately treated infection; her vomiting subsequently resolved.

Laboratory studies from the initial ED visit showed an A1c of 14%, blood glucose >450 mg/dL, normal white blood cell count, normal ESR, and CRP of 31 mg/L. Her diabetes was long-standing and uncontrolled due to medication nonadherence.

In the current presentation, the patient was alert but markedly disorganized, exhibiting confusion, disorientation, and fluent but nonsensical speech. Neurological examination revealed impaired comprehension with intact naming and repetition, without other focal neurological deficits. Vital signs, including blood pressure and heart rate, were normal and afebrile. Aside from severe hyperglycemia of blood glucose >400 mg/dL, laboratory studies were unremarkable.

Imaging

Because of concern for acute stroke, an initial CT angiogram of the head was obtained and demonstrated normal intracranial vasculature with no evidence of large vessel occlusion, perfusion abnormality, or acute ischemic changes. A subsequent non-contrast MRI of the brain revealed a T2 fluid-attenuated inversion recovery (FLAIR) hyperintensity in the left temporal lobe, prompting further evaluation with contrast-enhanced MRI. This demonstrated complete opacification of the left mastoid and middle ear cavity with a focal peripherally enhancing area concerning for coalescent mastoiditis and otitis media, along with erosion of the tegmen tympani mastoideum. A small, peripherally enhancing 9 × 5 × 7 mm collection was identified within the left middle temporal gyrus, consistent with a temporal lobe abscess, although assessment of restricted diffusion was limited by susceptibility artifact from the adjacent skull base. Following the MRI, a contrast-enhanced CT of the temporal bone was performed, which confirmed acute mastoiditis with otitis media; however, the temporal lobe abscess was better delineated on MRI, aligning with its superior sensitivity for detecting intracranial complications.

**Figure 1:**
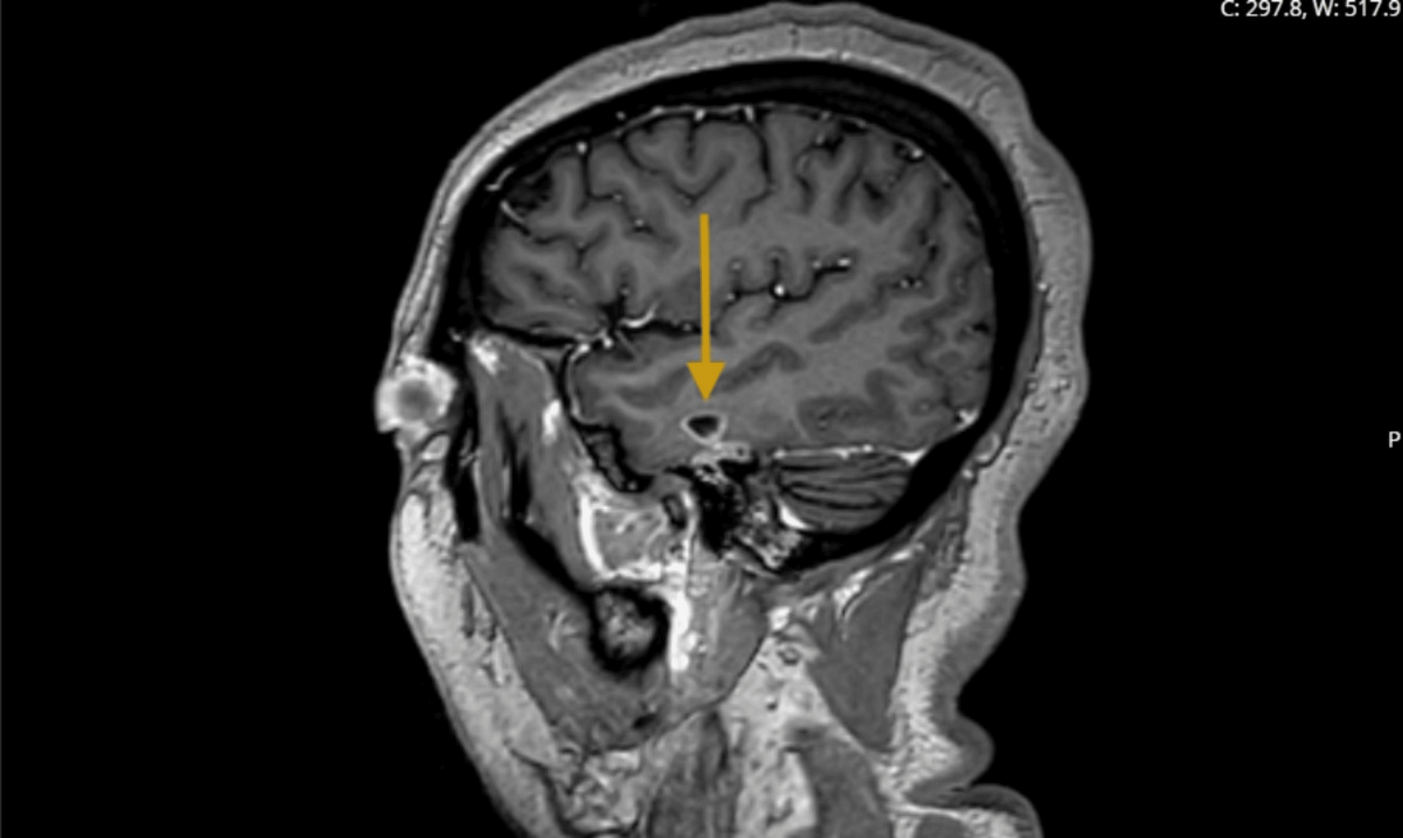
MRI of the brain with contrast (sagittal view) reveals a well-circumscribed, peripherally enhancing lesion within the left temporal lobe, corresponding to the measured 9 × 5 × 7 mm abscess.

**Figure 2:**
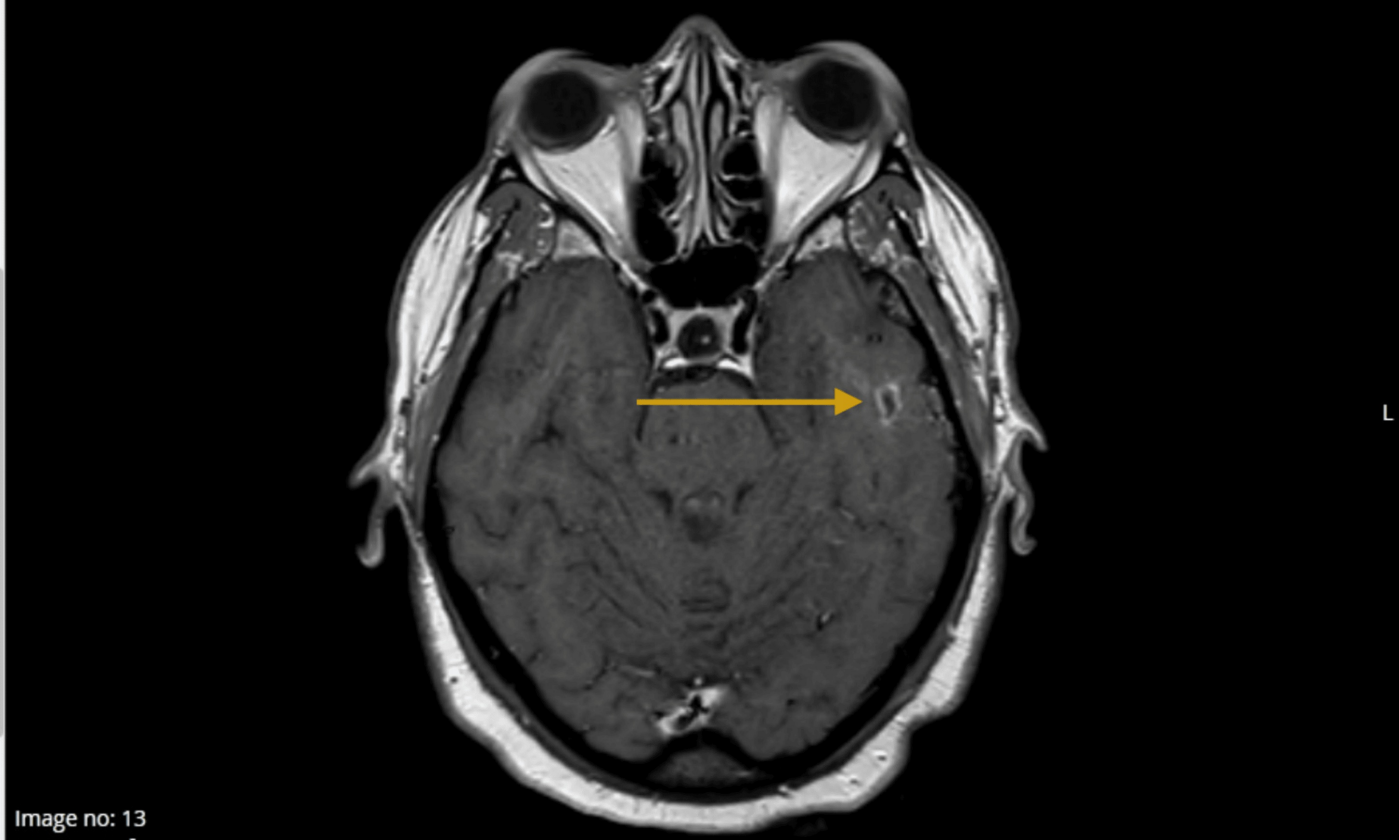
MRI of the brain with contrast MRI brain with contrast (coronal view) demonstrating a small peripherally enhancing fluid collection in the left temporal lobe (measuring 9 × 5 × 7 mm), consistent with a temporal lobe abscess.

Figure 1 (sagittal post-contrast MRI) and Figure 2 (coronal post-contrast MRI) depict the peripherally enhancing left temporal lobe abscess, demonstrating its morphology, extent, and relationship to adjacent structures.

Management

Neurologic evaluation attributed the patient’s altered mentation and fluent, nonsensical speech to involvement of the dominant temporal lobe, consistent with Wernicke’s aphasia. A multidisciplinary team-including neurology, neurosurgery, otolaryngology, neuroradiology, and infectious disease, collaboratively managed presumed mastoiditis complicated by intracranial abscess formation. Blood cultures were obtained prior to initiating therapy and were negative.

Empiric antimicrobial therapy was initiated with intravenous cefepime 2000 mg every 8 hours, vancomycin 1250 mg every 12 hours, and oral metronidazole 500 mg every 8 hours. This regimen was selected to provide broad coverage of organisms associated with otogenic intracranial infections, including *Pseudomonas aeruginosa*, which is more common in patients with uncontrolled diabetes and in cases of inadequately treated otitis externa. Oral metronidazole was chosen due to its high oral bioavailability, achieving serum concentrations comparable to intravenous dosing.

During admission, her diabetes was actively managed with insulin therapy and medication optimization to achieve appropriate glycemic control, addressing her longstanding nonadherence. After detailed imaging review, nonoperative management was recommended given the small abscess size, lack of significant mass effect, and favorable early clinical trajectory. A six-week antimicrobial course was planned, and a repeat MRI was scheduled for eight weeks to confirm radiographic resolution and guide subsequent care.

Outcome

Eight-week surveillance MRI demonstrated near-complete resolution of both the mastoiditis and intracranial abscess, with only minimal residual FLAIR signal in the left temporal lobe. The patient’s comprehension deficits, fluent but nonsensical speech, and overall confusion improved progressively, and her neurological function returned to baseline in parallel with radiographic resolution. She remained clinically stable with no recurrent otologic or neurologic complaints at follow-up.

## Discussion

This case illustrates the rare progression of acute otitis externa to coalescent mastoiditis and a temporal lobe abscess in a patient with poorly controlled diabetes. Diabetes is a known risk factor for severe and atypically presenting infections due to impaired immune defenses, and hyperglycemia may contribute to subtle neurologic findings and rapid spread of infection [3,10].

Most otogenic brain abscesses arise from chronic otitis media or longstanding mastoid disease, making acute otitis externa an unusual source [5,6,9]. Although intracranial complications have been reported in necrotizing otitis externa, such occurrences are rare [4]. In this case, inadequate treatment allowed extension of infection into the middle ear and mastoid, progressing to coalescent mastoiditis. MRI demonstrated erosion of the tegmen tympani, permitting direct intracranial spread into the temporal lobe, a well-described mechanism of otogenic brain abscess formation [5,6,9].

The diagnostic sequence highlights the importance of early MRI. The patient’s CT angiogram was normal, consistent with CT’s limitations in detecting early cerebritis or small abscesses. MRI subsequently identified mastoid involvement, skull base erosion, and the temporal lobe abscess, consistent with imaging recommendations for suspected intracranial complications of inflammatory ear disease [1,2,7].

The patient’s complete recovery with medical therapy alone aligns with evidence supporting antibiotic management for small abscesses without mass effect or neurological deterioration [8]. Prior reports demonstrate worse outcomes and higher surgical rates in larger or delayed-diagnosis abscesses [5,6,9]. Early recognition, MRI evaluation, and coordinated multidisciplinary care were central to the favorable outcome in this case.

Overall, this case reinforces the increased susceptibility of diabetic patients to rapid infectious spread and underscores the need for prompt imaging and treatment adherence. It also adds to the limited literature demonstrating that acute otitis externa, when inadequately treated, can progress to mastoiditis and intracranial abscess formation through tegmen tympani erosion.

## Conclusions

This case highlights the possibility of a suspected otogenic source contributing to severe intracranial complications in high-risk individuals, particularly those with poorly controlled diabetes mellitus. Although a definitive causal relationship between acute otitis externa and intracranial abscess formation cannot be established in the absence of microbiologic confirmation or comprehensive exclusion of alternative diabetic differential diagnoses such as necrotizing otitis externa or concurrent otitis media, the imaging findings of coalescent mastoiditis, tegmen tympani erosion, and a contiguous temporal lobe abscess strongly suggest an otogenic pathway of spread. Early clinical recognition, prompt MRI evaluation, and timely initiation of appropriate antimicrobial therapy remain critical to mitigating morbidity. Primary care clinicians, including family physicians, are uniquely positioned to identify patients whose symptoms diverge from the expected course. Sustained vigilance, structured follow-up, and early escalation of care when symptoms persist or evolve are essential to preventing the progression of an initially benign external ear infection into a potentially life-threatening intracranial process.

## References

[1] Hussain JA, Singh L, Pugh G (2025). Unveiling the hidden culprit: a case of a temporal lobe abscess presenting with nonspecific symptoms. Cureus.

[2] Agarwal M, Juliano AF, Hagiwara M (2025). ACR Appropriateness Criteria® inflammatory ear disease. J Am Coll Radiol.

[3] Bodilsen J, Duerlund LS, Mariager T (2023). Clinical features and prognostic factors in adults with brain abscess. Brain.

[4] Demirci T, O'Brien S (2019). Complicated necrotizing otitis externa progressing to coalescent mastoiditis and temporal lobe abscess. Am J Med.

[5] Duarte MJ, Kozin ED, Barshak MB (2018). Otogenic brain abscesses: a systematic review. Laryngoscope Investig Otolaryngol.

[6] Sun J, Sun J (2014). Intracranial complications of chronic otitis media. Eur Arch Otorhinolaryngol.

[7] Szyfter W, Kruk-Zagajewska A, Borucki L, Bartochowska A (2012). Evolution in management of otogenic brain abscess. Otol Neurotol.

[8] Carpenter J, Stapleton S, Holliman R (2007). Retrospective analysis of 49 cases of brain abscess and review of the literature. Eur J Clin Microbiol Infect Dis.

[9] Migirov L, Duvdevani S, Kronenberg J (2005). Otogenic intracranial complications: a review of 28 cases. Acta Otolaryngol.

[10] Joshi N, Caputo GM, Weitekamp MR, Karchmer AW (1999). Infections in patients with diabetes mellitus. N Engl J Med.

